# Familial resemblance in dietary intake among singletons, twins, and spouses: a meta-analysis of family-based observations

**DOI:** 10.1186/s12889-024-20798-x

**Published:** 2024-11-29

**Authors:** Farshad Teymoori, Mahdi Akbarzadeh, Mostafa Norouzzadeh, Mitra Kazemi Jahromi, Hossein Farhadnejad, Niloufar Saber, Hamid Ahmadirad, Mina Jahangiri, Danial Habibi, Parisa Riahi, Ebrahim Mokhtari, Maryam Zarkesh, Maryam S. Daneshpour, Parvin Mirmiran, Mohammadreza Vafa

**Affiliations:** 1 Nutritional Sciences Research Center, Iran University of Medical Sciences, Tehran, Iran; 2 Department of Nutrition, School of Public Health, Iran University of Medical Sciences, Tehran, Iran; 3 Cellular and Molecular Endocrine Research Center, Research Institute for Endocrine Sciences, Shahid Beheshti University of Medical Sciences, Tehran, Iran; 4 Nutrition and Endocrine Research Center, Research Institute for Endocrine Sciences, Shahid Beheshti University of Medical Sciences, Tehran, Iran; 5 Endocrinology and Metabolism Research Center, Hormozgan University of Medical Sciences, Bandar Abbas, Iran; 6 Department of Biostatistics, Faculty of Medical Sciences, Tarbiat Modares University, Tehran, Iran; 7 Department of Biostatistics and Epidemiology, School of Public Health, Babol University of Medical Sciences, Babol, Iran; 8 Department of Nutrition, Faculty of Public Health, Iran University of Medical Sciences, Tehran, Iran

**Keywords:** Energy, Macronutrient, Food groups, Siblings, Spouse, Meta-analysis

## Abstract

**Background:**

Familial dietary intake can be influenced by both genetic and environmental factors; the current study aimed to examine the role of these two factors on dietary intake by investigating the resemblance in energy, nutrient, and food group intake among spouses and siblings in twin and family-based studies.

**Methods:**

The online literature databases, including PubMed, Scopus, Web of Science, and Cochrane Library were systematically searched up to September 2024. The pooled correlation coefficient (r) of studies was calculated using Fisher’s z and standard error (SE) of z’s of all studies and our final results were reported in six groups including non-twin siblings, monozygotic (MZ) twins, dizygotic (DZ) twins, all-twins, all siblings, and spouse.

**Results:**

Our findings on 30 observational studies indicate that siblings, especially MZ twins, tend to have similar dietary intake, with high correlations for vegetables (r: 0.59), red meat (r: 0.58), and fruits (r: 0.56). Among DZ twins, the lowest correlations were observed for eggs (r: 0.07), soft drinks (r: 0.14), and daily intake of simple carbohydrates (r: 0.17). DZ twins did not show significant differences in dietary resemblance compared to non-twin siblings. Among spouses, the highest correlations for dietary intake were found for polyunsaturated fats (r: 0.41), saturated fats (r: 0.40), and total fats (r: 0.39), while the lowest correlation was for protein intake (r: 0.24).

**Conclusions:**

While the greatest similarity in dietary intake was observed among MZ twins, no significant difference in dietary intake similarity was noted between DZ twins and non-twin siblings. Furthermore, spouses exhibited a significant degree of similarity in their dietary consumption. Therefore, dietary intake is shaped by a complex interplay of genetic and environmental factors, warranting further investigation to validate these observations.

**Supplementary Information:**

The online version contains supplementary material available at 10.1186/s12889-024-20798-x.

## Background

Since the late 1970s, there has been increasing interest in studying the influence of genetics and familial factors on dietary choices and nutrient preferences [1]. This interest has been fueled by the understanding that diet plays a crucial role in preventing chronic diseases like cardiovascular diseases [2]. Despite efforts to promote healthy eating habits, rates of chronic diseases continue to rise, underscoring the urgent need to understand the complex factors that shape dietary behavior [3].

Previous research has found similarities in nutrient intake among family members, such as spouses, siblings, and twins, but it is still unclear whether these similarities are due to genetic or environmental factors [4–6]. Evidence from twin research suggests that genetic factors play a role in shaping dietary patterns, with monozygotic (MZ) twins having more similar food intake than dizygotic (DZ) twins [1, 7]. Additionally, environmental factors may also influence dietary behavior and preferences over time [8]. However, the effect of a shared growth environment on food choices is inconclusive [4, 5, 9, 10].

Of note, understanding the correlation between spouses and siblings in dietary habits can provide valuable insights to develop interventions for improving family members’ diets and reducing the risk of diet-related diseases. Besides, it can help to identify genetic and environmental factors that contribute to individual differences in food intake and diet-related outcomes [11].

This meta-analysis examines the correlation between spouses and sibling pairs in dietary intake, including energy, nutrients, and food groups, and compares the correlations based on factors like family relationships, dietary assessment approach, and sample size. Additionally, the mean correlations for energy and macronutrient intake between siblings are compared, to address differences between study groups. The study results can contribute to the growing body of research on the complex interplay between genetic and environmental factors in shaping dietary behavior and offer insights useful for public health interventions promoting a healthy eating lifestyle.

## Materials and methods

### Systematic search

We systematically searched PubMed, Scopus, Web of Science, and Cochrane databases up to September 2024 to find potential eligible studies. In this case, we combined keywords related to energy, macronutrients and food intake, diet, family members, and correlation to find eligible observational studies. The complete search strategy in databases is indicated in (Supplementary Table 1). Additionally, a manual search was done in the reference list of related reviews, original studies, and the Google Scholar database. Two reviewers independently screened studies first by title and abstract and if necessary, by full-text reviewing. The protocol for the present systematic review and meta-analysis has been registered in PROSPERO (CRD42024587951).

### Eligibility criteria

Our eligibility criteria were observational studies, either cohort, cross-sectional, or case-control studies, assessing the familial correlation of energy, macronutrients, and food intake at a time point among populations with any age group. The types of familial relationships considered in this study were non-twin siblings, monozygotic (MZ) twins, dizygotic (DZ) twins, all-twins, all siblings, and spouses. Our exclusion criteria were (1) unhealthy populations like people with cancer or diabetes; (2) articles not in English; (3) studies didn’t report the sample size of each correlation; (4) studies investigating the correlation of parents’ knowledge and children’s intake, and (5) studies investigating dietary intake among infants, as the influence of environmental factors and individual preferences in dietary choices is limited within this demographic. This results in negligible variations or resemblances in dietary habits among family members, potentially introducing bias into the findings.

### Data extraction

Two reviewers extracted the following data independently and in duplicate after reviewing full texts: first author name, year of publication, studies region, sex, sibling’s age, dietary assessment method (either food frequency questionnaire (FFQ), dietary records, or recalls), the reported unit of dietary variable (e.g., kcal, kj, gr, percent of energy or serving/day), a correlation coefficient (r) and its related sample size. The r refers to Pearson or Spearman rank correlation coefficients and, in some cases, it refers to intra-class correlation coefficients, which can be considered close to correlation coefficients. Any disagreement was resolved by discussion.

### Quality assessment

Studies quality was assessed using the Newcastle - Ottawa quality assessment scale (NOS) for cross-sectional studies. Assessments were conducted in terms of the following: (1) selection (representativeness of the exposed sample, selection of the non-exposed sample, ascertainment of exposure); (2) compatibility (comparability of outcome groups based on design or analysis), and (3) outcome (assessment of outcome, the statistical test is appropriate). In this context, five, two, and three points were given to selection, comparability, and outcome respectively. This scale’s final score of 7 or more indicates good quality.

### Statistical analysis

First, we extracted relationship resemblance, as presented by r, and related sample sizes for energy, carbohydrate, simple carbohydrate, protein, fat, saturated fatty acid (SFA), polyunsaturated fatty acid (PUFA), cholesterol, alcohol, fiber, fruit, vegetable, potato, seafood, egg, meats, red meat, dairy, soft drink, and alcohol intake in spouses and siblings.

We converted Pearson’s, Spearman’s, or intra-class correlation coefficients to z’s using Fisher’s z transformation to obtain approximate normality and then calculated a mean and standard error (SE) of transformed correlation weighted by the sample sizes in the studies [12]. The pooled r of studies was calculated using Fisher’s z and SE of z’s of all studies using the random effect method and then transformed into r. In this context, *r* < 0.30, 0.30 ≤ *r* < 0.50, and *r* ≥ 50, are considered weak, moderate, and strong correlations respectively [13]. Heterogeneity, qualitatively assessed by I^2^ statistic and P heterogeneity < 0.05 considered significant.

We considered pooled r and 95% confidence interval (CI) for reporting our final results in six groups including non-twin siblings (brother-brother, sister-sister, and brother-sister or a combination of them), MZ twins, DZ twins, all-twins (MZ and DZ twins), all siblings (twin and non-twin siblings), and spouse. If a study, reports one variable in two pair groups (e.g., a study reports energy intake resemblance for brother-brother and sister-sister relationships), these two pair groups are considered separately as different studies and analyzed in the subgroup of siblings.

For macronutrients including carbohydrates, protein, and fat, we pooled correlations of two reported values, first only macronutrients reported as percent of energy intake, and second a combination of macronutrients reported as percent of energy and grams of intake. In the second analysis, if a study reports both grams of intake and percent of energy, we considered them as a different study and then analyzed them together. The potential publication bias was tested using Egger’s test, Begg’s test, and visual inception of funnel plots.

Regarding the significant heterogeneity observed for some dietary variables among different pairs, we decided to conduct a meta-regression analysis using Z and SE_Z_ statistics for only energy and macronutrient intake as main dietary components for finding the source of heterogeneity among included studies. Based on the characteristics of included studies, some variables were selected for assessment as sources of heterogeneity including the year of publication (before the year 2000, after the year 2000), sex (both sexes, sex-specific), sibling type (twins, non-twins), region (America, Europe, Asia, and Oceania), dietary assessment method (FFQ, dietary records or recalls), sample size (lower and larger than 500 individuals, lower and larger than 1000 individuals), and reported units of macronutrients (gram, percent), sibling’s age (younger than 18, older than 18 years old). For studies that reported the age range of their participants, the decision for determining the age category was made based on the age group that had the largest population if has been reported; or based on the calculation of an approximate mean of the study population based on the reported mean age among different subgroups and their sample size. Therefore, an unadjusted model of meta-regression was conducted for any included variable and as a result, beta coefficients, 95% CI, P-value, and τ 2 were reported for each variable. Additionally, we conducted an analysis of variance (ANOVA) test to compare the mean observed correlations for energy, carbohydrate, protein, and fat intake among siblings, MZ twins, and DZ twins, both overall and in pairs. The significant levels were considered as *P* < 0.05. Statistical analysis was done using MedCalc software (version 20.218, Ostend, Belgium; https://www.medcalc.org; 2023) and STATA software version 17.0.

## Results

### The literature searches

**Fig. 1:**
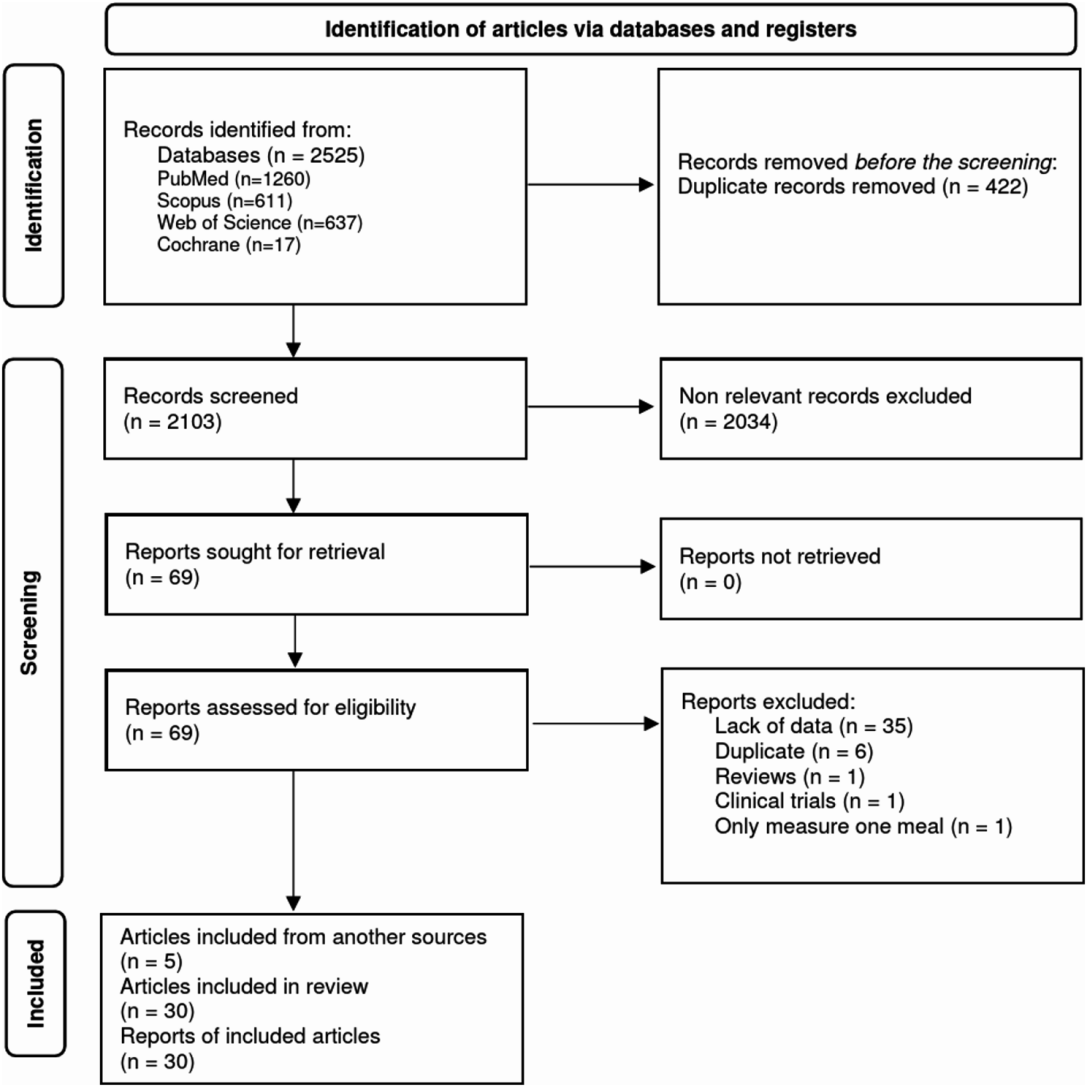
Flow diagram of selection of the published studies

Initially, 2,525 documents were retrieved. After removing 422 duplicates and excluding 2,034 irrelevant papers, 69 articles remained for abstract and full-text review. Of these, 44 articles were excluded based on the inclusion and exclusion criteria, leaving 25 articles. Additionally, 5 articles were sourced from other references, resulting in a total of 30 articles included in this meta-analysis (Fig. 1).

### Characteristics of included studies

The general characteristics of the 30 articles included in the study, all of which have a cross-sectional design, are provided in (Supplementary Table 2) [1, 4–7, 9, 11, 14–36]. These studies were performed in the USA (*n* = 12) [1, 9, 11, 14–16, 18–20, 26, 27, 29], Canada (*n* = 2) [7, 31], Finland (*n* = 2) [5, 34], Netherlands (*n* = 2) [23, 24], Korea (*n* = 2) [28, 32], China (*n* = 2) [33, 35], UK (*n* = 1) [4], Ireland (*n* = 1) [30], Sweden (*n* = 1) [25], Australia (*n* = 1) [17], Denmark (*n* = 1) [6], Norway (*n* = 1) [21], France (*n* = 1) [22] and Iran (*n* = 1) [36]. The papers were published between 1978 and 2023. The number of participants in the included studies assessed the dietary resemblance ranged from 42 to 9798 with an age range of 1 and 56 years. All included articles were high-quality based on the NOS scores (Supplementary Table 3). The correlation of different nutrients (in grams of intake or percent of energy or both of them) and food groups (in gram intake or serving) between 4 paired groups including MZ twins, DZ twins, siblings, and spouses is presented in (Supplementary Table 2).

The statistical distribution of included articles based on their characteristics is reported in (Table 1). Articles were classified based on sex (male, female, and both genders), year of publication (before 2000 and after 2000), region (America, Europe, and Asia-Oceania), dietary assessment method (24-h recalls or records, FFQ, and a mixed approach), siblings age (≤ 18 years, ˃18 years, and spouse), sample size (≤ 500, 500–1000, and ˃1000).

**Table 1:** Statistical distribution of included studies based on their characteristics in both absolute and proportional terms

	69 extracted Studies	30 included Papers
**Sex** (***n*/percent)**		
M	14/20	-
F	13/19	*-*
Both	42/61	-
**Year of publication (n/percent)**		
Before 2000	-	15/50
2000 and after	-	15/50
**Region (n/percent)**		
America	-	14/47
Europe	-	10/33
Asia and Oceania	-	6/20
**Dietary assessment method (n/percent)**		
24-h recalls or records	-	12/40
FFQ	-	15/50
Mix	-	3/10
**Siblings age (n/percent)**		
≤ 18 years	-	12/40
> 18 years	-	12/40
NA (Spouse)	-	6/20
**Sample size (n/percent)**		
≤ 500	-	10/33
500–1000	-	7/23
> 1000	-	13/44

*Abbreviations*: M, Male; F, Female; MZ Twins, Monozygotic twins; DZ Twins, Dizygotic twins; FFQ, Food frequency questionnaire; NA, Not applicable

### Meta-analysis

**Fig. 2:**
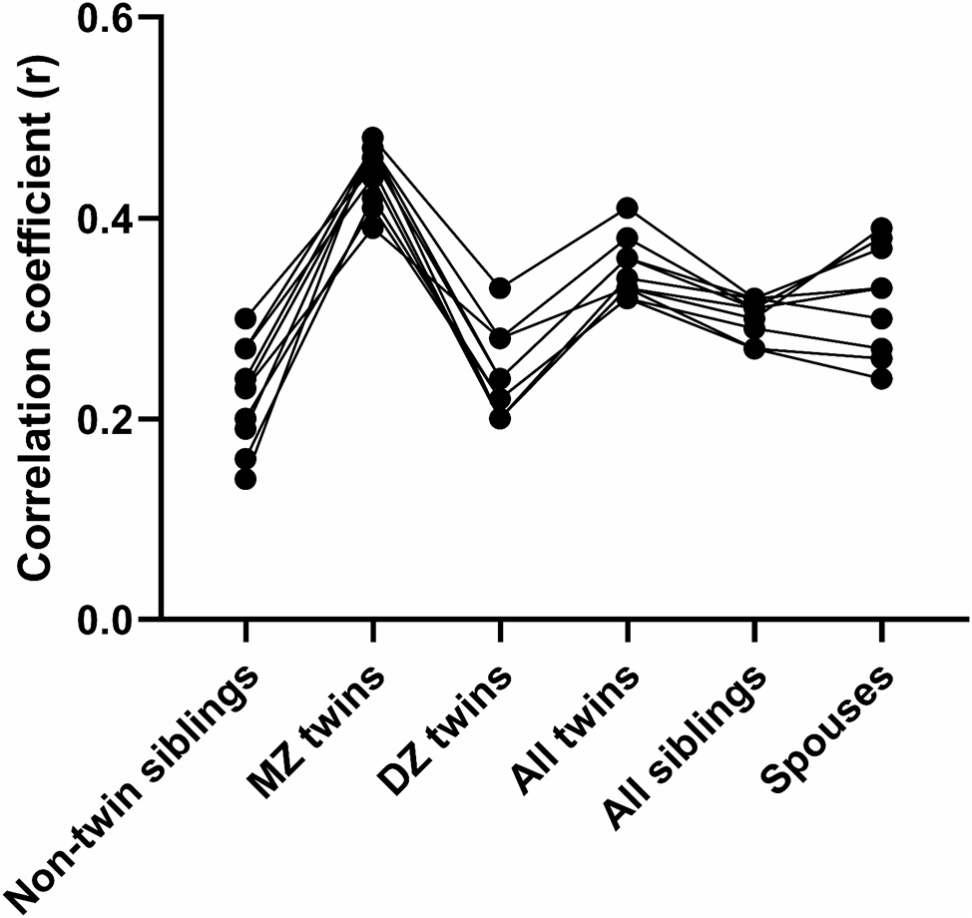
Familial resemblance of energy and macronutrient intake among non-twin siblings, monozygotic twins, dizygotic twins, all twins, all siblings, and spouses

**Fig. 3:**
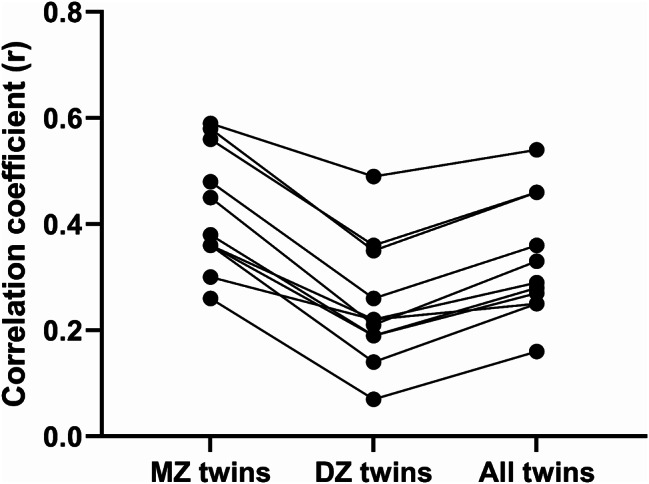
Familial resemblance of food group intake among monozygotic twins, dizygotic twins, and all twins

(Figs. 2 and 3and Supplementary Table 2) shows the pooled results of the meta-analysis on dietary factors correlation among non-twin siblings, MZ twins, DZ twins, all twins, all siblings including twins and non-twins, and spouses. In non-twin siblings, the highest pooled r value was reported for PUFA (0.32), and fat (percent of energy intake) (0.30). In MZ twins, the highest pooled r value was reported for food groups including vegetable (0.59), red meat (0.58), and fruit (0.56). Also, in DZ twins the highest pooled r value was reported for vegetables (0.49), fruit (0.36), and red meat (0.35). In spouses, the highest pooled r was related to PUFA (0.41), and SFA (0.40), cholesterol (0.39), fat (total) (0.39), and alcohol (0.38), and total carbohydrate (percent of energy intake) (0.38).

The publication bias assessment using the P-value of the Egger test indicated that there was significant publication bias among sibling subsets for energy, carbohydrate, protein, and alcohol intake. Also, in the twin subgroup, we observed a significant publication bias for protein, fat, vegetable, and potato intake. In addition, in the spouse subgroup, a significant publication bias was identified for fat, SFA, and PUFA intake (Supplementary Figs. 1–48).

Also, there was significant heterogeneity in the meta-analysis of dietary factors and different familial groups. (Table 2) indicated the meta-regression to find the potential sources of heterogeneity in all sibling and spouse groups. None of the potential sources of heterogeneity had a significant association with energy and fat intake in all siblings. A significant and inverse association was observed for the type of relationship (non-twin siblings versus twins) with carbohydrate and protein intake. Also, among spouses, the year of publication showed an inverse association with energy, fat, carbohydrate, and protein intake. Moreover, the dietary assessment method was a source of heterogeneity, as other methods (dietary records or recalls) had more estimates of energy, carbohydrate, and protein intake than FFQ.

**Table 2:** Meta-regression analysis for energy and macronutrient intake in spouse and all siblings

Siblings - unadjusted model					Spouse - unadjusted model				
**Variable**	**Coefficient**	**95% CI**	**P-value**	**τ 2**	**Variable**	**Coefficient**	**95% CI**	**P-value**	**τ 2**
**Energy**				0.041	**Energy**				0.008
Year^1^	-0.09	-0.21, 0.02	0.13	0.026	Year^1^	**-0.16**	**-0.28**,** -0.03**	**0.01**	**0.003**
Sex^2^	0.07	-0.06, 0.20	0.28	0.027					
Relationship^3^	-0.04	-0.18, 0.08	0.45	0.028					
Region^4^	0.07	-0.07, 0.21	0.32	0.028	Region^5^	-0.04	-0.18, 0.10	0.53	0.008
Dietary Assessment^6^	0.04	-0.06, 0.18	0.35	0.027	Dietary Assessment^6^	**0.14**	**0.02**,** 0.25**	**0.02**	**0.004**
Child age^7^	-0.12	-0.24, 0.00	0.06	0.025					
Sample size^8^	-0.08	-0.20, 0.04	0.18	0.026	Sample size^9^	0.03	-0.11, 0.17	0.65	0.009
**Fat**				0.045	**Fat**				0.027
Year^1^	-0.05	-0.16, 0.05	0.34	0.031	Year^1^	**-0.21**	**-0.43**,** -0.00**	**0.04**	**0.020**
Sex^2^	-0.00	-0.12, 0.11	0.93	0.032					
Relationship^3^	-0.09	-0.20, 0.01	0.09	0.030					
Region^5^	0.06	-0.04, 0.17	0.26	0.031	Region^5^	0.09	-0.09, 0.29	0.30	0.027
Dietary Assessment^6^	0.04	-0.06, 0.15	0.44	0.031	Dietary Assessment^6^	0.12	-0.06, 0.31	0.17	0.025
Child age^7^	-0.05	-0.17, 0.05	0.29	0.031					
Sample size^8^	-0.01	-0.12, 0.09	0.80	0.032	Sample size^9^	0.16	-0.01, 0.34	0.06	0.022
Variable unites^10^	-0.03	-0.14, 0.08	0.58	0.032	Variable unites^10^	-0.03	-0.23, 0.17	0.75	0.029
**Carbohydrate**				0.037	**Carbohydrate**				0.012
Year^1^	-0.03	-0.16, 0.08	0.55	0.030	Year^1^	**-0.18**	**-0.33**,** -0.04**	**0.01**	**0.004**
Sex^2^	-0.00	-0.12, 0.12	0.98	0.030					
Relationship^3^	**-0.20**	-0.31, -0.10	**0.00**	0.019					
Region^4^	0.03	-0.08, 0.15	0.58	0.030	Region^5^	-0.11	-0.30, 0.07	0.20	0.009
Dietary Assessment^6^	0.04	-0.08, 0.16	0.49	0.030	Dietary Assessment^6^	**0.18**	**0.03**,** 0.33**	**0.02**	**0.005**
Child age^7^	-0.21	-0.14, 0.10	0.72	0.030					
Sample size^8^	-0.10	-0.22, 0.00	0.06	0.027	Sample size^9^	0.05	-0.15, 0.26	0.55	0.014
Variable unites^10^	-0.03	-0.16, 0.08	0.51	0.030	Variable unites^10^	0.09	-0.11, 0.29	0.32	0.012
**Protein**				0.031	**Protein**				0.030
Year^1^	-0.08	-0.19, 0.02	0.14	0.022	Year^1^	**-0.32**	**-0.44**,** -0.19**	**0.001**	**0.004**
Sex^2^	0.00	-0.10, 0.11	0.95	0.024					
Relationship^3^	**-0.14**	-0.24, -0.04	**0.00**	0.019					
Region^4^	0.01	-0.09, 0.12	0.75	0.024	Region^5^	-0.14	-0.40, 0.11	0.23	0.027
Dietary Assessment^6^	0.08	-0.02, 0.19	0.12	0.023	Dietary Assessment^6^	**0.26**	**0.06**,** 0.46**	**0.01**	**0.015**
Child age^7^	-0.00	-0.11, 0.10	0.90	0.024					
Sample size^8^	-0.09	-0.10, 0.00	0.06	0.021	Sample size^9^	0.008	-0.27, 0.29	0.94	0.034
Variable unites^10^	-0.05	-0.16, 0.05	0.31	0.023	Variable unites^10^	-0.03	-0.32, 0.25	0.77	0.034

^1^Published paper after 2000 versus before 2000

^2^ Studies work on both sexes versus studies work on specific sex (male or female)

^3^ Not Twin siblings versus twins

^4^ Others (Asia and Oceania) versus Europe and America

^5^ Others (Europe, Asia, and Oceania) versus America

^6^ Dietary intakes assessed by records or recall versus FFQ

^7^ Children older than 18 years old versus younger

^8^ Study population larger than 1000 people versus lower than 1000 people

^9^ Study population larger than 500 people versus lower than 500 people

^10^ Units of variable (percent of energy versus gram of intake)

**NOTE.** Significant coefficients are bolded

(Table 3) shows the mean r difference of energy and macronutrient intake in twins and siblings. The findings indicated the mean r difference among all siblings-MZ twins and MZ twins-DZ twins was significant in energy and macronutrients (P-value < 0.05). This difference remained significant in the comparisons between MZ twins and non-twin siblings, as well as MZ and DZ twins, but except for carbohydrates, the similarity of energy, fat, and protein intake did not differ between non-twin siblings and DZ twins.

**Table 3:** Twin and siblings mean r differences in energy and macronutrients*

Nutrients	Mean *r*			Overall and pairwise *P*-values			
	1-non twin Sib	2-MZ	3-DZ	P_ANOVA_	P_1 − 2_	P_1 − 3_	P_2 − 3_
Energy	0.25	0.47	0.21	0.001	0.002	0.506	0.001
CHO	0.16	0.46	0.29	0.001	0.001	0.024	0.003
Protein	0.19	0.38	0.26	0.007	0.002	0.225	0.032
Fat	0.23	0.45	0.21	0.001	0.001	0.745	0.001

**Abbreviations: Sib**, Siblings; **MZ**, Monozygotic twins; **DZ**, Dizygotic twins; **CHO**, Carbohydrate

* Significance level considered P-value < 0.05

## Discussion

This meta-analysis is the first to assess familial resemblance in dietary intakes across different family pairs (siblings, twins, and spouses), considering covariates such as gender, study timing and location, dietary measurement method, dietary variable units, sample size, and age. Overall, our study indicated that sibling pairs exhibit a weak to strong similarity in dietary intake. However, this resemblance is more pronounced among twin pairs. Notably, while there was a significant difference in energy and macronutrient intake between MZ twins and non-twin sibling pairs, this difference is not observed between DZ twins and non-twin sibling pairs. Additionally, spouses also demonstrate similarities in dietary intake, which can sometimes be comparable to those observed among siblings and even twins.

Our findings suggest that MZ twins exhibit the highest familial dietary resemblance, likely due to their close genetic similarity, which contributes to similar dietary intakes and patterns established during childhood [37]. These similarities may stem from both shared genetic factors among biologically related relatives and shared environmental factors among MZ twins living in the same household [4, 6]. However, these genetic effects can sometimes outweigh environmental influences, as studies on twins have shown that heritability and familial resemblance in dietary intakes are stronger in twins with closer genetic characteristics compared to those observed in broader family-based studies [5].

In our analysis of DZ twins, we observed a lower resemblance in dietary intake compared to MZ twins. Furthermore, the similarity in dietary intake between DZ twins and non-twin siblings did not differ significantly. The nearly identical genetic makeup of MZ twins likely accounts for their shared food preferences and dietary habits. In contrast, DZ twins, who have more varied genetic compositions, may show greater variation in dietary intake, food choices, tastes, and preferences [10]. Additionally, the high genetic similarity of MZ twins may indirectly lead to greater similarity in food choices and nutritional intake compared to DZ twins by influencing factors such as the childhood family environment, upbringing, social interactions with common friends, and other related aspects [10].

Our results also indicate a weak to moderate resemblance in dietary intake among sibling pairs. A previous meta-analysis by Wang et al. reviewed 24 studies focusing on dietary intakes among parent-child pairs [13]. The findings from Wang’s study revealed a weak similarity in dietary intake between parents and their children [13]. This conclusion was further supported by Parvin et al. in 2023, which also confirmed the weak resemblance in dietary intakes among parent-child pairs [38]. The meta-analysis by Teymoori et al. [39], which considered a broader range of food items and parent-child relationships, found that dietary intake similarities among parent-child pairs were generally weak, with the strongest resemblance observed in mother-daughter pairs. These findings align with our study in two key ways. First, mother-daughter pairs may exhibit greater similarity potentially due to their closer genetic relationship. Additionally, the lifestyle similarities between mothers and daughters, along with shared environmental factors, could further explain the stronger resemblance in dietary intake between these pairs [39].

Furthermore, our results indicate that siblings sometimes exhibit stronger and, at other times, weaker similarities in nutritional intake compared to spouses. In other words, the degree of dietary similarity among siblings varies depending on their genetic resemblance, while among spouses, it is more influenced by shared environmental factors. However, these genetic and environmental influences are not fixed. In certain instances, the similarities in food intake among siblings may surpass those observed in spouses. This could be attributed to environmental factors, such as school meal programs in some countries, where children consume most of their daily meals, including breakfast and lunch, at school. This shared experience could be a source of dietary similarity among siblings [40, 41]. Additionally, spouses have increasingly shifted from consuming traditional family foods to eating a wider variety of foods outside the home, such as at restaurants and workplaces. Meanwhile, children may become more autonomous in their food choices when regularly eating without their parents [42]. The modern lifestyle, characterized by a lack of quality family time, can impact the eating behaviors of both spouses and siblings, leading to fewer shared family meals, more frequent dining out, and greater reliance on takeaways or home delivery [43, 44]. However, in some instances, spouses exhibit greater similarity in dietary intake than sibling pairs. This could be attributed to a more similar dietary composition between spouses, as well as a tendency in some societies for spouses to control their total energy intake due to concerns about weight gain [13].

The meta-regression in the current study revealed that the approach used to assess dietary intake was a significant source of heterogeneity in the study results. Studies that utilized dietary recalls or records demonstrated higher similarities in energy and macronutrient intakes compared to those using FFQ. This difference may be because dietary records or recalls are often conducted on the same days, leading to stronger correlations. In contrast, FFQs assess the frequency of a broader range of foods over a longer period which may result in lower correlations [45, 46]. Additionally, these results may be influenced by biases inherent in different dietary assessment methods, leading to varying levels of dietary intake misreporting [38]. While correlations greater than 0.5 are generally considered strong [13], these biases in nutritional evaluations may necessitate lowering this threshold for categorizing weak, moderate, and strong correlations. However, further studies are needed to confirm this adjustment. Furthermore, the publication year of the studies contributed to heterogeneity in our results. In this meta-analysis, studies published after 2000 showed lower similarities in dietary intake compared to those conducted before 2000. In recent decades, there has been an increase in food and beverage consumption outside the home [47]. Additionally, the development of the food industry has led to a wider variety of products, preparation methods, and cooking styles, making a broader range of foods more accessible [48]. The nutritional transition has also significantly altered food culture, shifting from traditional, stable dietary patterns to increased consumption of fast foods, often consumed outside the home [47, 49].

Future research could explore the heritability of dietary intakes among family members. Additionally, examining the resemblance of dietary intake through dietary patterns like the Healthy Eating Index (HEI) and Diet Quality Index (DQI) could serve as a key objective for future studies. Several questions remain unresolved in this area, such as how the type of nutritional assessment tool and dietary information source among family members influence dietary intake resemblance. Also, the priority and contribution of each of the genetic and environmental factors on food intake is not yet known.

The strengths of this meta-analysis are worth highlighting. Our study is the first comprehensive meta-analysis to assess familial resemblance among family pairs. Additionally, the eligible studies analyzed were conducted in diverse populations with varying demographic, socioeconomic, and nutritional characteristics, allowing the findings on familial resemblance and dietary intake correlations to be broadly generalized across different populations. This meta-analysis has also several limitations. First, we encountered challenges in examining the correlation of all dietary items across study groups, as some available studies focused solely on twins (MZ and DZ), with no studies conducted on spouses, all-sibling groups, or non-twin siblings. Additionally, meta-regression analysis revealed heterogeneity in the results of studies examining dietary intake correlations among spouses and siblings. Key sources of this heterogeneity included family relationship type, publication year, and dietary assessment method. This variability made it difficult to reach a definitive conclusion across the studies. Another limitation is the presence of publication bias for some variables, suggesting the potential absence of relevant studies in this field.

## Conclusions

Our results indicate that siblings may exhibit similarities in dietary intake. Greater genetic similarity, such as in MZ twins, tends to enhance these dietary similarities. However, the influence of environmental factors is evident, as DZ twins, despite their more genetic resemblance, did not show significant differences in dietary intake compared to non-twin siblings. Although spouses share fewer genetic similarities than siblings, their shared living environment often results in dietary intake similar to those of sibling pairs. While further studies are necessary to confirm or refute these findings, it appears that dietary intake is shaped by a complex interaction of genetic and environmental factors.

## Electronic supplementary material

Below is the link to the electronic supplementary material.

Supplementary Material 1

Supplementary Material 2

Supplementary Material 3

Supplementary Material 4

Supplementary Material 5

Supplementary Material 6

Supplementary Material 7

Supplementary Material 8

Supplementary Material 9

Supplementary Material 10

Supplementary Material 11

Supplementary Material 12

Supplementary Material 13

Supplementary Material 14

Supplementary Material 15

Supplementary Material 16

Supplementary Material 17

Supplementary Material 18

Supplementary Material 19

Supplementary Material 20

Supplementary Material 21

Supplementary Material 22

Supplementary Material 23

Supplementary Material 24

Supplementary Material 25

Supplementary Material 26

Supplementary Material 27

Supplementary Material 28

Supplementary Material 29

Supplementary Material 30

Supplementary Material 31

Supplementary Material 32

Supplementary Material 33

Supplementary Material 34

Supplementary Material 35

Supplementary Material 36

Supplementary Material 37

Supplementary Material 38

Supplementary Material 39

Supplementary Material 40

Supplementary Material 41

Supplementary Material 42

Supplementary Material 43

Supplementary Material 44

Supplementary Material 45

Supplementary Material 46

Supplementary Material 47

Supplementary Material 48

Supplementary Material 49

Supplementary Material 50

Supplementary Material 51

Supplementary Material 52

Supplementary Material 53

## Data Availability

The data used and/ or analyzed in the present study are available from the corresponding author upon reasonable request.
