# Supplementary material for: Familial resemblance in dietary intake among singletons, twins, and spouses: a meta-analysis of family-based observations
Source: BMC Public Health. 2024 Nov 29;24:3328. doi: 10.1186/s12889-024-20798-x (PMC11605858; doi:10.1186/s12889-024-20798-x)
Supplement: Supplementary file 1 — Supplementary Material 1 [file 12889_2024_20798_MOESM1_ESM.docx]

| **Supplementary Table 1.** Search strategy to find potential eligible studies. | | |
| --- | --- | --- |
| **Databases** | **Search Strategy** | **Studies count (n)** |
| **PubMed** | (("resemblanc*"[Title/Abstract] OR "aggregat*"[Title/Abstract] OR "correlat*"[Title]) AND ("Fathers"[MeSH Terms] OR "Mothers"[MeSH Terms] OR "Parents"[MeSH Terms] OR "Family"[MeSH Terms] OR "famil*"[Title] OR "Adolescent"[MeSH Terms] OR "Child"[MeSH Terms] OR "Twins"[MeSH Terms] OR "twin*"[Title] OR "Siblings"[MeSH Terms] OR "dyad*"[Title] OR "offspring*"[Title] OR "pair*"[Title]) AND ("Food"[MeSH Terms] OR "Diet"[MeSH Terms])) | 1260 |
| **Scopus** | (TITLE (nutrient* OR intak* OR pattern* OR food* OR diet*) AND TITLE (father* OR mother* OR parent* OR famil* OR adolescent* OR child* OR twin* OR dyad* OR sibling* OR pair* OR offspring*) AND TITLE (resemblanc* OR aggregat* OR correlat*)) | 611 |
| **Web of Science** | (TI=(father* OR mother* OR parent* OR famil* OR dyad* OR twin* OR offspring* OR pair* OR sibling* OR adolescen* OR child)) AND (TI=( resemblanc* OR aggregat* OR correlat* )) AND (TI=( nutrient* OR intak* OR pattern* OR food* OR diet*)) | 637 |
