# Supplementary material for: Familial resemblance in dietary intake among singletons, twins, and spouses: a meta-analysis of family-based observations
Source: BMC Public Health. 2024 Nov 29;24:3328. doi: 10.1186/s12889-024-20798-x (PMC11605858; doi:10.1186/s12889-024-20798-x)
Supplement: Supplementary file 2 — Supplementary Material 2 [file 12889_2024_20798_MOESM2_ESM.docx]

| **Supplementary Table 2.** Population characteristics of included studies assessed the dietary resemblance. | | | | | | | | |
| --- | --- | --- | --- | --- | --- | --- | --- | --- |
| **Author, year, (quality score^1^)** | **Country** | **SS** | **Siblings age**  **(Year)** | **Dietary assessment** | **MZ** | **DZ** | **Non-twin Sib** | **Spouse** |
| Fabsitz, 1978  (8/10) | USA | MZ: 464  DZ: 446 | 42-56 | FFQ | Energy: 0.50  G: (CHO: 0.43, Simple CHO: 0.32, Pro: 0.44, Fat: 0.42, SFA: 0.45, PUFA: 0.27, Alc: 0.42)  Serving/day: (Fruit: 0.19, Potatoes: 0.33, Egg: 0.22, Red meat: 0.24, Dairy: 0.25) | Energy: 0.11  G: (CHO: 0.12, Simple CHO: 0.12, Pro: 0.09, Fat: 0.14, SFA: 0.15, PUFA: 0.1, Alc: 0.2)  Serving/day: (Fruit: 0.21, Potatoes: 0.19, Egg: 0.17, Red meat: 0.15, Dairy: 0.19) | - | - |
| Lee, 1982  (8/10) | USA | Spouse: 2856 | NA (Spouses) | Food questionnaire | - | - | - | Energy: 0.35  G: (CHO: 0.35, Pro: 0.38, Fat: 0.46, Chl: 0.35) |
| Rozin, 1987  (8/10) | USA | MZ: 24  DZ: 18 | 17-26 | Questionnaire and phone interview | Serving/day: (Egg: 0.04) | Serving/day: (Egg: 0.46) | - | - |
| Perruse, 1988  (9/10) | Canada | Sib: 361  MZ: 60  DZ: 60  Spouse: 678 | 14.8 | 3-d dietary record | Energy: 0.65  G: (CHO: 0.68, Pro: 0.65, Fat: 0.60)  P: (CHO: 0.70, Pro: 0.71, Fat: 0.61) | Energy: 0.48  G: (CHO: 0.45, Pro: 0.42, Fat: 0.55)  P: (CHO: 0.49, Pro: 0.55, Fat: 0.59) | Energy: 0.23  G: (CHO: 0.27, Pro: 0.25, Fat: 0.23)  P: (CHO: 0.37, Pro: 0.38, Fat: 0.36) | Energy: 0.37  G: (CHO: 0.37, Pro: 0.47, Fat: 0.37)  P: (CHO: 0.5, Pro: 0.28, Fat: 0.45) |
| Patterson, 1988  (9/10) | USA | Sib_1_: 132  Sib_2_: 169  Spouse_1_: 145  Spouse_1_: 143 | 11.8 | FFQ (36 items), 24h recall, and 3-day food diaries | - | - | Energy _1_: 0.48  Energy _2_: 0.38  G_1_: (Fat: 0.06, SFA: 0.1)  G_2_: (Fat: 0.23, SFA: 0. 26) | Energy _1_: 0.44  Energy _2_: 0.28  G_1_: (Fat: 0.44, SFA: 0.2)  G_2_: (Fat: 0.14, SFA: 0. 44) |
| Heller, 1988  (9/10) | Australia | MZ: 212  DZ: 188 | 36.2 | 4-day weighed food diaries | Energy: 0.45  P: (CHO: 0.47, Simple CHO: 0.28, Pro: 0.43, Fat: 0.23, SFA: 0.15, PUFA: 0.21, Alc: 0.34) | Energy: 0.26  P: (CHO: 0.31, Simple CHO: 0.18, Pro: 0.38, Fat: 0.11, SFA: 0.09, PUFA: 0.19, Alc: 0.23) | - | - |
| Sellers, 1991  (8/10) | USA | Sib: 650 | Adult Man siblings | Burke Diet History | - | - | Energy: 0.09  G: (CHO: 0.01, Pro: 0.21, Fat: 0.13, SFA: 0.11, PUFA: 0.11, Alc: 0.07, Chl: 0.09)  P: (CHO: 0.00, Pro: 0.00, Fat: 0.04, SFA: 0.06, PUFA: 0.01) | - |
| Oliveria, 1992  (7/10) | USA | Spouse: 166 | NA (Spouses) | 3-d dietary records | - | - | - | Energy: 0.39  G: (CHO: 0.44, Pro: 0.38, Fat: 0.39, SFA: 0.56, PUFA: 0.38, Chl: 0.44) |
| De castro, 1993 (9/10) | USA | MZ: 218  DZ: 172 | 38.8 | 7-day food intake diaries | Energy: 0.63  G: (CHO: 0.59, Pro: 0.54, Fat: 0.47, Alc: 0.52) | Energy: 0.42  G: (CHO: 0.44, Pro: 0.42, Fat: 0.32, Alc: 0.26) | - | - |
| Rossow, 1994 (8/10) | Norway | Spouse: 544 | NA (Spouses) | Questionnaire | - | - | - | G: (Fat: 0.61, Alc: 0.66) |
| Vauthier, 1996 (9/10) | France | Sib: 774  Spouse: 387 | 14.2 | 3-day food consumption diaries | - | - | Energy: 0.37  P: (CHO: 0.41, Pro: 0.46, Fat: 0.46) | Energy: 0.38  P: (CHO: 0.36, Pro: 0.47, Fat: 0.37) |
| Feunekes, 1997 (7/10) | Netherlands | Sib: 1541  Spouse: 941 | 1-30 (averaged^2^) | 2-day diet records | - | - | Energy: 0.55  G: (Fat: 0.61, SFA: 0.62, PUFA: 0.61, Chl: 0.61)  P: (Fat: 0.65, SFA: 0.63, PUFA: 0.63, Chl: 0.58) | G: (Fat: 0.58, SFA: 0.57, PUFA: 0.59, Chl: 0.55)  P: (Fat: 0.54, SFA: 0.54, PUFA: 0.61, Chl: 0.51) |
| Hur, 1998  (9/10) | USA | MZ: 132  DZ: 102  Spouse: 66 | 42.4 | FFQ (67 items) | Energy: 0.23  G: (CHO: 0.14, Simple CHO: 0.1, Pro: 0.08, Fat: 0.28, SFA: 0.32, PUFA: 0.35, Alc: 0.34)  Serving/day: (Dairy: 0.33, Soft drink: 0.17) | Energy: 0.33  G: (CHO: 0.36, Simple CHO: 0.3, Pro: 0.59, Fat: 0.39, SFA: 0.33, PUFA: 0.44, Alc: 0.44)  Serving/day: (Dairy: 0.09, Soft drink: 0.17) | - | Energy: 0.3  G: (CHO: 0.18, Simple CHO: 0.04, Pro: 0.22, Fat: 0.36, SFA: 0.37, PUFA: 0.01, Alc: 0.34) |
| Feunekes, 1998 (9/10) | Netherlands | Spouse: 253 | NA (Spouses) | Self-administered FFQ | - | - | - | Energy: 0.23  P: (Fat: 0.3, SFA: 0.34, PUFA: 0.34, Chl: 0.51) |
| Heitmann, 1999  (9/10) | Sweden | MZM: 106  DZM: 146  MZF: 90  DZF: 206 | 46 | Questionnaire | **MZM**  Serving/day: (Fruit: 0.43, Vegetable: 0.28, Seafood: 0.03, Egg: 0.11, Meats: 0.33, Dairy: 0.41)  **MZF**  Serving/day: (Fruit: 0.2, Vegetable: 0.41, Seafood: 0.16, Egg: 0.05, Meats: 0.02, Dairy: -0.08) | **DZM**  Serving/day: (Fruit: 0.09, Vegetable: 0.10, Seafood: 0.03, Egg: 0.1, Meats: 0.18, Dairy: -0.11)  **DZF**  Serving/day: (Fruit: -0.08, Vegetable: 0.05, Seafood: -0.07, Egg: -0.15, Meats: 0.32, Dairy: -0.06) | - | - |
| McCaffery, 2001 (7/10) | USA | MZ: 174  DZ: 134 | 20.8 | 3-day dietary records | Energy: 0.38  G: (Fat: 0.26) | Energy: 0.08  G: (Fat: 0.18) | - | - |
| Mitchell, 2003 (8/10) | USA | Sib: 1147  Spouse: 128 | > 16 | FFQ (102 items) | - | - | Energy: 0.09  G: (CHO: 0.07, SFA: 0.1, Alc: 0.09)  P: (Pro: 0.1, Fat: 0.04, Chl: 0.06) | Energy: 0.27  G: (CHO: 0.22, Pro: 0.10, SFA: 0.20)  P: (Fat: 0.14, Chl: 0.24, Alc: 0.04) |
| Park, 2004  (9/10) | Korea | Spouse: 260 | NA (Spouses) | FFQ | - | - | - | Energy: 0.25  G: (SFA: 0.26, Chl: 0.31)  P: (CHO: 0.26, Pro: 0.07, Fat: 0.4) |
| Breen, 2006  (9/10) | UK | MZ: 206  DZ: 222 | 4.4 | FFQ (95 items) | Serving/day: (Vegetable: 0.82) | Serving/day: (Vegetable: 0.59) | - | - |
| Hasselbalch, 2008  (9/10) | Denmark | MZM: 224  MZF: 244  DZM: 236  DZF: 248  DZOS: 248 | 38 | FFQ (247 items) | **MZM**  Energy: 0.43  P: (CHO: 0.4, Pro: 0.25, Fat: 0.38, Alc: 0.47, Fiber: 0.46)  Serving/day: (Fruit: 0.49, Vegetable: 0.65, Potatoes: 0.67, Seafood: 0.55, Egg: 0.1, Red meat: 0.34, Dairy: 0.34, Soft drink: 0.29)  **MZF**  Energy: 0.32  P: (CHO: 0.49, Pro: 0.52, Fat: 0.53, Alc: 0.6, Fiber: 0.37)  Serving/day: (Fruit: 0.51, Vegetable: 0.64, Potatoes: 0.61, Seafood: 0.63, Egg: 0.51, Red meat: 0.29, Dairy: 0.36, Soft drink: 0.4) | **DZM**  Energy: 0.07  P: (CHO: 0.14, Pro: 0.18, Fat: 0.09, Alc: 0.21, Fiber: 0.13)  Serving/day: (Fruit: 0.38, Vegetable: 0.48, Potatoes: 0.43 Seafood: 0.51, Egg: 0.08, Red meat: 0.19, Dairy: 0.19, Soft drink: 0.01)  **DZF**  Energy: 0.02  P: (CHO: 0.12, Pro: 0.07, Fat: 0.13, Alc: 0.18, Fiber: 0.29, Vegetable: 0.51)  Serving/day: (Fruit: 0.26, Potatoes: 0.44, Seafood: 0.34, Egg: 0.13, Red meat: 0.19, Dairy: 0.16, Soft drink: 0.15)  **DZOS**  Energy: 0.11  P: (CHO: 0.22, Pro: 0.04, Fat: 0.13, Alc: 0.09, Fiber: 0.08)  Serving/day: (Fruit: 0.48, Vegetable: 0.13, Seafood: 0.15, Egg: -0.07, Red meat: 0.09, Dairy: -0.01, Soft drink: -0.01) | - | - |
| Faith, 2008  (7/10) | \|  \| \| --- \| \|  \| \|  \| \| USA \| \|  \| \|  \| \|  \| \|  \| | MZM: 204  DZM: 192  MZF: 224  DZF: 172 | 7.47 | 24-h dietary recalls | **MZM**  Serving/day: (Fruit: 0.88, Vegetable: 0.85, Red meat: 0.89)  **MZF**  Serving/day: (Fruit: 0.81, Vegetable: 0.83, Red meat: 0.77) | **DZM**  Serving/day: (Fruit: 0.75, Vegetable: 0.72, Red meat: 0.61)  **DZF**  Serving/day: (Fruit: -0.79, Vegetable: 0.88, Red meat: 0.71) | - | - |
| Keskitalo, 2008  (9/10) | Finland | MZM: 826  DZM: 720  MZF: 500  DZF: 616  DZOS: 1356 | 24.4 | FFQ | **MZM**  Serving/day: (Fruit: 0.55, Vegetable: 0.46, Potatoes: 0.41, Seafood: 0.52, Egg: 0.34, Meats: 0.23)  **MZF**  Serving/day: (Fruit: 0.52, Vegetable: 0.49, Potatoes: 0.39, Seafood: 0.44, Egg: 0.44, Meats: 0.42) | **DZM**  Serving/day: (Fruit: 0.26, Vegetable: 0.23, Potatoes: 0.27, Seafood: 0.2, Egg: 0.16, Meats: 0.17)  **DZF**  Serving/day: (Fruit: -0.29, Vegetable: 0.26, Potatoes: 0.23, Egg: 0.08, Meats: 0.32)  **DZOS**  Serving/day: (Fruit: -0.16, Vegetable: 0.12, Potatoes: 0.1, Seafood: 0.12, Egg: 0.09, Meats: 0.11) | - | - |
| Shrivastava, 2012 (8/10) | Ireland | Spouse: 1450 | NA (Spouses) | FFQ (149 items) | - | - | - | Energy: 0.19  G: (CHO: 0.19, Pro: 0.12, Fat: 0.17) |
| Liu, 2013  (9/10) | USA | MZM: 186  DZM: 92  MZF: 190  DZF: 104  DZOS: 144 | 11.7 | 3-day food intake diaries | **MZM**  Energy: 0.45  G: (CHO: 0.36, Pro: 0.29, Fat: 0.46)  **MZF**  Energy: 0.60  G: (CHO: 0.55, Pro: 0.37, Fat: 0.53) | **DZM**  Energy: 0.06  G: (CHO: 0.16, Pro: 0.12, Fat: 0.19)  **DZF**  Energy: 0.08  G: (CHO: 0.33, Pro: 0.16, Fat: 0.12)  **DZOS**  Energy: 0.08  G: (CHO: 0.14, Pro: -0.04, Fat: 0.09) |  |  |
| Dubois, 2013  (9/10) | Canada | MZ: 308  DZ: 430 | 9 | Two multiple passes 24-h dietary recalls | Energy: 0.43  G: (CHO: 0.42, Pro: 0.36, Fat: 0.36)  P: (CHO: 0.28, Simple CHO: 0.3, Pro: 0.18, Fat: 0.29) | Energy: 0.21  G: (CHO: 0.23, Pro: 0.17, Fat: 0.15)  P: (CHO: 0.18, Pro: 0.21, Fat: 0.16) | - | - |
| Lee, 2015  (9/10) | Korea | Sib: 1969  Spouse: 2341 | 10.7 | FFQ and 24-h recall | - | - | G: (Pro: 0.23) | G: (Pro: 0.06) |
| Li, 2016  (9/10) | China | MZM: 144  DZM: 62  MZF: 148  DZF: 84 | 11.6 | FFQ (145 items) | **MZM**  Energy: 0.61  G: (CHO: 0.64, Pro: 0.52, Fat: 0.61, Fruit: 0.26, Vegetable: 0.36, Seafood: 0.16, Egg: 0.0, Meats: 0.5, Dairy: 0.52)  P: (CHO: 0.46, Pro: 0.26, Fat: 0.52)  M: (Soft drink: 0.69)  **MZF**  Energy: 0.47  G: (CHO: 0.46, Pro: 0.51, Fat: 0.62, Fruit: 0.40, Vegetable: 0.29, Seafood: 0.19, Egg: 0.51, Meats: 0.55, Dairy: 0.02)  P: (CHO: 0.55, Pro: 0.43, Fat: 0.61)  M: (Soft drink: 0.17) | **DZM**  Energy: 0.52  G: (CHO: 0.59, Pro: 0.39, Fat: 0.09, Fruit: 0.19, Vegetable: 0.65, Seafood: 0.4, Egg: 0.03, Meats: 0.19, Dairy: 0.12)  P: (CHO: 0.18, Pro: 0.68, Fat: 0.0)  M: (Soft drink: 0.1)  **DZF**  Energy: 0.4  G: (CHO: 0.56, Pro: 0.52, Fat: 0.54, Fruit: 0.08, Vegetable: 0.83, Seafood: 0.02, Egg: 0.36, Meats: 0.31, Dairy: 0.91)  P: (CHO: 0.49, Pro: 0.37, Fat: 0.52)  M: (Soft drink: 0.5) | - | - |
| Bogl, 2017  (9/10) | Finland | Sib: 750 | 11 | 24-h dietary recalls | - | - | Energy: 0.28  P: (CHO: 0.31, Simple CHO: 0.34, Pro: 0.31, Fat: 0.32) | - |
| Huang, 2017  (9/10) | China | MZ: 324  DZ: 298 | 11.3 | FFQ (145 items) | P: (Fat: 0.48) | P: (Fat: 0.22) | - | - |
| Teymoori 2023  (8/10) | Iran | BB:  BS:  SiSi: | >18 | FFQ | - | - | Energy: (BB: 0.10, BS: 0.05, SiSi: 0.21)  G-CHO: (BB: 0.14, BS: 0.03, SiSi: 0.21)  P-CHO: (BB: 0.12, BS: 0.07, SiSi: 0.18)  G-Fat: (BB: 0.08, BS: 0.02, SiSi: 0.03)  P-Fat: (BB: 0.17, BS: 0.10, SiSi: 0.19)  G-Pro: (BB: 0.13, BS: 0.06, SiSi: 0.17)  P-Pro: (BB: 0.11, BS: 0.09, SiSi: 0.16) | - |
| **Abbreviations:** **Alc,** Alcohol; **BB,** Brother-Brother; **BS,** Brother-Sister; **Chl,** Cholesterol, **CHO,** Carbohydrate; **DZ,** Dizygotic twins; **DZF,** DZ Female; **DZM,** DZ Male; **FFQ,** Food Frequency Questionnaire; **G**, Gram; **M,** Milliliter; **MZ,** Monozygotic twins; **MZF,** MZ Female; **MZM,** MZ Male; **NA**, Not Applicable; **P,** Percent of energy, **Pro,** Protein;**PUFA,** Poly Unsaturated Fatty Acid; **SFA,** Saturated Fatty Acid; **Sib,** Sibling; **SiSi,** Sister-Sister; **SS,** Sample Size.  ^1^ Based on Newcastle-Ottawa Scale (NOS)  ^2^ Since most of the population is under 18 years old (only 6% of the study population was older than 21), this study is considered under 18 years old. | | | | | | | | |
