# Supplementary material for: Familial resemblance in dietary intake among singletons, twins, and spouses: a meta-analysis of family-based observations
Source: BMC Public Health. 2024 Nov 29;24:3328. doi: 10.1186/s12889-024-20798-x (PMC11605858; doi:10.1186/s12889-024-20798-x)
Supplement: Supplementary file 3 — Supplementary Material 3 [file 12889_2024_20798_MOESM3_ESM.docx]

| **Supplementary Table 3.** Quality Assessment using Newcastle-Ottawa Scale (NOS) for Cross-sectional studies^1^ | | | | | | | |
| --- | --- | --- | --- | --- | --- | --- | --- |
|  | **Selection** | | | **Comparability** | **Outcome** | | Study  score |
| Study | Representativeness of the exposed sample | Selection of the non-exposed sample | Ascertainment of exposure | Comparability of Outcome groups on the  Basis of design or analysis | Assessment of outcome | The statistical test is appropriate |  |
| Park et al, 2004 | * | ** | ** | * | ** | * | 9/10 |
| Lee et al, 2015 | * | ** | ** | * | ** | * | 9/10 |
| Hasselbalch, A.2008 | * | ** | ** | * | ** | * | 9/10 |
| Mitchell, B.2003 | - | ** | ** | * | ** | * | 8/10 |
| Huang, T.2017 | * | ** | ** | * | ** | * | 9/10 |
| Li, J.2016 | * | ** | ** | * | ** | * | 9/10 |
| Fabsitz, R.1977 | - | ** | ** | * | ** | * | 8/10 |
| Hur, Y.1998 | * | ** | ** | * | ** | * | 9/10 |
| Heller, R.1988 | * | ** | ** | * | ** | * | 9/10 |
| Faith, M.2008 | * | * | * | * | ** | * | 7/10 |
| Perusse, L.1988 | * | ** | ** | * | ** | * | 9/10 |
| Dubois, L.2013 | * | ** | ** | * | ** | * | 9/10 |
| Vauthier et al, 1996 | * | ** | ** | * | ** | * | 9/10 |
| De Castro et al, 1992 | * | ** | ** | * | ** | * | 9/10 |
| Rozin et al, 1987 | * | ** | * | * | ** | * | 8/10 |
| Breen et al, 2006 | * | ** | ** | * | ** | * | 9/10 |
| Heitmann et al, 1999 | * | ** | ** | * | ** | * | 9/10 |
| Keskitalo et al, 2008 | * | ** | ** | * | ** | * | 9/10 |
| McCaffery et al, 2001 | * | * | * | * | ** | * | 7/10 |
| Sellers et al, 1991 | * | ** | * | * | ** | * | 8/10 |
| Bogl et al, 2017 | * | ** | ** | * | ** | * | 9/10 |
| Feunekes et al, 1997 | * | ** | * | * | ** | * | 8/10 |
| Feunekes et al, 1998 | * | ** | ** | * | ** | * | 9/10 |
| Lee et al, 1982 | * | ** | * | * | ** | * | 8/10 |
| Pattrson et al, 1988 | * | ** | ** | * | ** | * | 9/10 |
| Rossow et al, 1994 | * | ** | * | * | ** | * | 8/10 |
| Oliveria et al, 1992 | - | ** | * | * | ** | * | 7/10 |
| Shrivastava et al, 2013 | * | ** | * | * | ** | * | 8/10 |
| Liu et al, 2013 | * | ** | * | * | ** | * | 8/10 |
| Teymoori 2023 | * | ** | * | * | ** | * | 8/10 |
| ^1^We used a modified NOS scale for cross-sectional studies. Note: A score of 7 or higher was considered a good study. | | | | | | | |
