# Supplementary material for: Familial resemblance in dietary intake among singletons, twins, and spouses: a meta-analysis of family-based observations
Source: BMC Public Health. 2024 Nov 29;24:3328. doi: 10.1186/s12889-024-20798-x (PMC11605858; doi:10.1186/s12889-024-20798-x)
Supplement: Supplementary file 4 — Supplementary Material 4 [file 12889_2024_20798_MOESM4_ESM.docx]

| **Supplementary Table 4.** Pooled results of spouses and sibling correlations of energy and nutrient intakes, by type of various pairs. | | | | | | |
| --- | --- | --- | --- | --- | --- | --- |
|  | **Non-twin Sib** | **MZ** | **DZ** | **All. Twins** | **All. Sib** | **Spouse** |
| **Energy (n)^1^** | 7/10 | 10/13 | 10/15 | 10/28 | 17/39 | 10/12 |
| Range | 0.09, 0.55 | 0.23, 0.65 | 0.02, 0.52 | 0.02, 0.65 | 0.02, 0.65 | 0.19, 0.47 |
| *Pooled r* (95% CI)^2^ | 0.27 (0.15, 0.39) | 0.47 (0.41, 0.53) | 0.20 (0.12, 0.27) | 0.34 (0.27, 0.41) | 0.32 (0.25, 0.38) | 0.33 (0.27, 0.39) |
| Heterogeneity (%) ^3^ | 97.8 | 76.2 | 74.4 | 89.4 | 94.8 | 84.7 |
| Eager (P-value) ^4^ | 0.32 | 0.55 | 0.08 | 0.47 | **0.02** | 0.98 |
| **Total carbohydrate^5^ (n)** | 5/11 | 9/12 | 9/14 | 9/26 | 14/45 | 8/9 |
| Range | 0.07, 0.41 | 0.14, 0.68 | 0.12, 0.59 | 0.12, 0.68 | 0.00, 0.68 | 0.18, 0.50 |
| *Pooled r* (95% CI)^2^ | 0.19 (0.12, 0.26) | 0.47 (0.40, 0.53) | 0.28 (0.20, 0.36) | 0.38 (0.31, 0.44) | 0.31 (0.26, 0.36) | 0.33 (0.25, 0.40) |
| Heterogeneity (%) ^3^ | 94.6 | 76.8 | 76.1 | 86.4 | 93.4 | 88.9 |
| Eager (P-value) ^4^ | **0.01** | 0.41 | **0.01** | 0.09 | **0.01** | 0.95 |
| **Total carbohydrate^6^ (n)** | 4/6 | 5/7 | 5/8 | 5/15 | 9/22 | 3/3 |
| Range | 0.07, 0.41 | 0.28, 0.70 | 0.12, 0.49 | 0.12, 0.70 | 0.00, 0.55 | 0.26, 0.5 |
| *Pooled r* (95% CI)^2^ | 0.24 (0.13, 0.34) | 0.47 (0.38, 0.55) | 0.24 (0.16, 0.33) | 0.36 (0.28, 0.44) | 0.31 (0.24, 0.37) | 0.38 (0.23, 0.51) |
| Heterogeneity (%) ^3^ | **95.8** | 75.2 | 65.4 | 84.4 | 92.8 | 88.4 |
| Eager (P-value) ^4^ | **0.01** | **0.01** | 0.06 | **0.03** | **0.01** | 0.05 |
| **Total carbohydrate^7^ (n)** | 3/5 | 7/9 | 7/10 | 7/19 | 11/25 | 6/6 |
| Range | 0.03, 0.2 | 0.14, 0.68 | 0.12, 0.59 | 0.12, 0.68 | 0.01, 0.68 | 0.18, 0.44 |
| *Pooled r* (95% CI)^2^ | 0.14 (0.05, 0.22) | 0.48 (0.38, 0.56) | 0.33 (0.22, 0.43) | 0.41 (0.32, 0.48) | 0.32 (0.25, 0.39) | 0.30 (0.21, 0.38) |
| Heterogeneity (%) ^3^ | 92.0 | 82.5 | 80.2 | 86.9 | 94.6 | 87.1 |
| Eager (P-value) ^4^ | 0.27 | 0.48 | **0.02** | 0.12 | **0.01** | 0.86 |
| **Simple carbohydrate (n)** | - | 3/3 | 3/3 | 3/6 | 4/7 | - |
| Range | - | 0.10, 032 | 0.12, 0.30 | 0.10, 0.30 | 0.10, 0.34 | - |
| *Pooled r* (95% CI)^2^ | - | 0.25 (0.13, 0.36) | 0.17 (0.07, 0.26) | 0.21 (0.13, 0.30) | 0.24 (0.16, 0.32) | - |
| Heterogeneity (%) ^3^ | - | **62.9** | **33.0** | 64.1 | 73.0 | - |
| Eager (P-value) ^4^ | - | 0.26 | 0.12 | 0.89 | 0.34 | - |
| **Protein^5^ (n)** | 6/12 | 9/12 | 9/14 | 9/26 | 15/46 | 9/10 |
| Range | 0.06, 0.46 | 0.08, 0.65 | -0.04, 0.5 | -0.04, 0.65 | -0.04, 0.68 | 0.06, 0.47 |
| *Pooled r* (95% CI)^2^ | 0.20 (0.14, 0.26) | 0.41 (0.34, 0.48) | 0.22 (0.14, 0.30) | 0.32 (0.24, 0.38) | 0.27 (0.23, 0.31) | 0.26 (0.14, 0.37) |
| Heterogeneity (%) ^3^ | 94.3 | 78.7 | 75.8 | 86.9 | 91.3 | 96.5 |
| Eager (P-value) ^4^ | 0.01 | 0.78 | 0.09 | 0.29 | **0.01** | 0.80 |
| **Protein^6^ (n)** | 5/7 | 5/7 | 5/8 | 5/13 | 10/23 | 4/4 |
| Range | 0.09, 0.46 | 0.18, 0.71 | 0.04, 0.68 | 0.04, 0.71 | 0.00, 0.71 | 0.07, 0.47 |
| *Pooled r* (95% CI)^2^ | 0.23 (0.13, 0.32) | 0.39 (0.25, 0.50) | 0.28 (0.14, 0.41) | 0.33 (0.23, 0.42) | 0.27 (0.20, 0.34) | 0.24 (0.05, 0.41) |
| Heterogeneity (%) ^3^ | 95.9 | 71.6 | 72.5 | 74.4 | 90.9 | 91.5 |
| Eager (P-value) ^4^ | **0.03** | 0.15 | **0.03** | **0.01** | **0.01** | 0.54 |
| **Protein^7^ (n)** | 3/5 | 7/9 | 7/10 | 7/19 | 11/25 | 6/6 |
| Range | 0.06, 0.25 | 0.08, 0.65 | -0.04, 0.50 | -0.04, 0.65 | -0.04, 0.65 | 0.06, 0.47 |
| *Pooled r* (95% CI)^2^ | 0.16 (0.09, 0.23) | 0.42 (0.33, 0.50) | 0.22 (0.14, 0.30) | 0.32 (0.24, 0.38) | 0.29 (0.23, 0.34) | 0.27 (0.11, 0.42) |
| Heterogeneity (%) ^3^ | 90.5 | 79.7 | 75.8 | 86.9 | 90.6 | 97.7 |
| Eager (P-value) ^4^ | 0.35 | 0.76 | 0.09 | 0.29 | **0.01** | 0.78 |
| **Fat^5^ (n)** | 7/17 | 11/14 | 11/16 | 11/30 | 18/53 | 12/15 |
| Range | 0.04, 0.65 | 0.23, 0.62 | 0.09, 0.55 | 0.09, 0.62 | 0.00, 0.65 | 0.14, 0.61 |
| *Pooled r* (95% CI)^2^ | 0.27 (0.14, 0.38) | 0.44 (0.38, 0.50) | 0.20 (0.14, 0.26) | 0.33 (0.26, 0.39) | 0.30 (0.25, 0.36) | 0.39 (0.31, 0.47) |
| Heterogeneity (%) ^3^ | 98.6 | 77.9 | 64.4 | 86.8 | 96.2 | 94.5 |
| Eager (P-value) ^4^ | 0.5 | 0.23 | **0.04** | 0.28 | 0.13 | 0.55 |
| **Fat^6^ (n)** | 6/8 | 6/8 | 6/9 | 6/17 | 12/25 | 6/6 |
| Range | 0.04, 0.65 | 0.23, 0.61 | 0.00, 0.59 | 0.00, 0.61 | 0.00, 0.65 | 0.14, 0.54 |
| *Pooled r* (95% CI)^2^ | 0.30 (0.12, 0.45) | 0.45 (0.35, 0.54) | 0.20 (0.11, 0.30) | 0.33 (0.24, 0.42) | 0.31 (0.22, 0.39) | 0.38 (0.28, 0.47) |
| Heterogeneity (%) ^3^ | 98.8 | 82.1 | 76.0 | 88.8 | 96.8 | 87.5 |
| Eager (P-value) ^4^ | 0.46 | 0.23 | 0.16 | 0.15 | 0.30 | **0.01** |
| **Fat^7^ (n)** | 7/7 | 8/10 | 8/11 | 8/21 | 13/29 | 7/8 |
| Range | 0.06, 0.61 | 0.26, 0.60 | 0.09, 0.55 | 0.09, 0.62 | 0.06, 0.62 | 0.14, 0.58 |
| *Pooled r* (95% CI)^2^ | 0.23 (0.03, 0.41) | 0.46 (0.38, 0.53) | 0.24 (0.15, 0.33) | 0.36 (0.28, 0.43) | 0.32 (0.24, 0.39) | 0.37 (0.24, 0.48) |
| Heterogeneity (%) ^3^ | 97.5 | 78.5 | 72.2 | 86.0 | 95.5 | 95.9 |
| Eager (P-value) ^4^ | **0.04** | 0.14 | **0.03** | 0.23 | 0.18 | 0.73 |
| **SFA (n)** | 4/11 | 3/3 | 3/3 | 3/6 | 8/19 | 7/9 |
| Range | 0.08, 0.63 | 0.15, 0.45 | 0.09, 0.33 | 0.09, 0.45 | 0.20, 0.57 | 0.20, 0.57 |
| *Pooled r* (95% CI)^2^ | 0.24 (0.10, 0.38) | 0.31 (0.11, 0.49) | 0.17 (0.05, 0.28) | 0.25 (0.11, 0.38) | 0.23 (0.12, 0.33) | 0.40 (0.29, 0.49) |
| Heterogeneity (%) ^3^ | 98.9 | 87.7 | **52.8** | 86.6 | 98.2 | 89.4 |
| Eager (P-value) ^4^ | 0.70 | 0.50 | 0.61 | 0.70 | 0.84 | **0.02** |
| **PUFA (n)** | 2/8 | 3/3 | 3/3 | 3/6 | 6/16 | 4/5 |
| Range | 0.13, 0.69 | 0.21, 0.35 | 0.10, 0.44 | 0.10, 0.44 | 0.06, 0.63 | 0.01, 0.59 |
| *Pooled r* (95% CI)^2^ | 0.32 (0.14, 0.47) | 0.26 (0.20, 0.33) | 0.23 (0.04, 0.40) | 0.24 (0.15, 0.33) | 0.26 (0.15, 0.36) | 0.41 (0.27, 0.53) |
| Heterogeneity (%) ^3^ | 99.2 | **0.00** | 82.2 | 70.4 | 98.1 | 91.6 |
| Eager (P-value) ^4^ | 0.20 | 0.79 | 0.18 | 0.18 | 0.93 | **0.01** |
| **Cholesterol (n)** | - | - | - | - | 3/3 | 6/6 |
| Range | - | - | - | - | 0.06, 0.61 | 0.24, 0.55 |
| *Pooled r* (95% CI)^2^ | - | - | - | - | 0.27 (-0.16, 0.62) | 0.39 (0.29, 0.49) |
| Heterogeneity (%) ^3^ | - | - | - | - | 99.41 | 90.55 |
| Eager (P-value) ^4^ | - | - | - | - | 0.49 | 0.84 |
| **Alcohol (n)** | - | 5/6 | 5/7 | 5/13 | 7/15 | 3/3 |
| Range | - | 0.34, 0.60 | 0.09, 0.44 | 0.09, 0.60 | 0.07, 0.60 | 0.04, 0.66 |
| *Pooled r* (95% CI)^2^ | - | 0.45 (0.37, 0.53) | 0.21 (0.15, 0.28) | 0.33 (0.24, 0.42) | 0.30 (0.20, 0.39) | 0.38 (-0.11, 0.72) |
| Heterogeneity (%) ^3^ | - | 0.92 | **46.3** | 86.9 | 91.8 | 96.8 |
| Eager (P-value) ^4^ | - | 0.92 | 0.12 | 0.75 | **0.02** | 0.37 |
| **Fiber (n)** | - | 2/3 | 2/4 | 2/7 | - | - |
| Range | - | 0.32, 0.46 | 0.08, 0.29 | 0.08, 0.46 | - | - |
| *Pooled r* (95% CI)^2^ | - | 0.38 (0.30, 0.46) | 0.19 (0.08, 0.30) | 0.28 (0.17, 0.37) | - | - |
| Heterogeneity (%) ^3^ | - | **34.9** | 65.9 | 79.2 | - | - |
| Eager (P-value) ^4^ | - | 0.87 | 0.52 | 0.54 | - | - |
| **Fruit (n)** | - | 7/12 | 7/14 | 7/26 | - | - |
| Range | - | 0.19, 0.88 | -0.08, 0.79 | -0.08, 0.88 | - | - |
| *Pooled r* (95% CI)^2^ | - | 0.56 (0.40, 0.69) | 0.36 (0.22,.48) | 0.46 (0.35, 0.56) | - | - |
| Heterogeneity (%) ^3^ | - | 97.3 | 96.0 | 97.3 | - | - |
| Eager (P-value) ^4^ | - | 0.71 | 0.19 | 0.13 | - | - |
| **Vegetable (n)** | - | 6/11 | 6/13 | 6/24 | - | - |
| Range | - | 0.28, 0.85 | 0.05, 0.88 | 0.05, 0.88 | - | - |
| *Pooled r* (95% CI)^2^ | - | 0.59 (0.45, 0.71) | 0.49 (0.33, 0.62) | 0.54 (0.43, 0.63) | - | - |
| Heterogeneity (%) ^3^ | - | 96.2 | 97.3 | 97.4 | - | - |
| Eager (P-value) ^4^ | - | 0.59 | **0.009** | **0.007** | - | - |
| **Potatoes (n)** | - | 3/5 | 3/7 | 3/12 | - | - |
| Range | - | 0.33, 0.67 | 0.10, 0.44 | 0.10, 0.67 | - | - |
| *Pooled r* (95% CI)^2^ | - | 0.48 (0.36, 0.59) | 0.26 (0.16, 0.35) | 0.36 (0.26, 0.45) | - | - |
| Heterogeneity (%) ^3^ | - | 91.9 | 89.6 | 94.4 | - | - |
| Eager (P-value) ^4^ | - | 0.10 | 0.09 | **0.02** | - | - |
| **Seafood (n)** | - | 4/8 | 4/10 | 4/18 | - | - |
| Range | - | 0.03, 0.63 | -0.07, 0.51 | -0.07, 0.63 | - | - |
| *Pooled r* (95% CI)^2^ | - | 0.36 (0.22, 0.49) | 0.19 (0.10, 0.29) | 0.27 (0.17, 0.37) | - | - |
| Heterogeneity (%) ^3^ | - | 91.7 | 87.1 | 93.9 | - | - |
| Eager (P-value) ^4^ | - | 0.06 | 0.64 | 0.94 | - | - |
| **Egg (n)** | - | 6/10 | 6/12 | 6/22 | - | - |
| Range | - | 0.03, 0.51 | -0.15, 0.46 | -0.15, 0.51 | - | - |
| *Pooled r* (95% CI)^2^ | - | 0.26 (0.14, 0.36) | 0.07 (0.01, 0.14) | 0.16 (0.08, 0.24) | - | - |
| Heterogeneity (%) ^3^ | - | 88.4 | 73.3 | 90.0 | - | - |
| Eager (P-value) ^4^ | - | 0.25 | 0.93 | 0.82 | - | - |
| **Meats (n)** | - | 3/6 | 3/7 | 3/13 | - | - |
| Range | - | 0.05, 0.55 | 0.11, 0.32 | 0.05, 0.55 | - | - |
| *Pooled r* (95% CI)^2^ | - | 0.36 (0.22, 0.48) | 0.22 (0.14, 0.30) | 0.29 (0.20, 0.36) | - | - |
| Heterogeneity (%) ^3^ | - | 87.3 | 77.4 | 87.2 | - | - |
| Eager (P-value) ^4^ | - | 0.56 | 0.28 | 0.14 | - | - |
| **Red meat (n)** | - | 3/5 | 3/6 | 3/11 | - | - |
| Range | - | 0.24, 0.89 | 0.09, 0.71 | 0.09, 0.89 | - | - |
| *Pooled r* (95% CI)^2^ | - | 0.58 (0.21, 0.80) | 0.35 (0.12, 0.54) | 0.46 (0.25, 0.63) | - | - |
| Heterogeneity (%) ^3^ | - | 98.5 | 95.6 | 97.7 | - | - |
| Eager (P-value) ^4^ | - | 0.23 | 0.11 | 0.05 | - | - |
| **Dairy (n)** | - | 5/8 | 5/9 | 5/17 | - | - |
| Range | - | -0.08, 0.52 | -0.11, 0.91 | -0.11, 0.91 | - | - |
| *Pooled r* (95% CI)^2^ | - | 0.30 (0.20, 0.39) | 0.22 (-0.01, 0.42) | 0.25 (0.13, 0.37) | - | - |
| Heterogeneity (%) ^3^ | - | 76.3 | 95.5 | 93.0 | - | - |
| Eager (P-value) ^4^ | - | 0.99 | 0.41 | 0.40 | - | - |
| **Soft drink (n)** | - | 3/5 | 3/6 | 3/11 | - | - |
| Range | - | 0.17, 0.69 | -0.01, 0.50 | -0.01, 0.69 | - | - |
| *Pooled r* (95% CI)^2^ | - | 0.36 (0.15, 0.54) | 0.14 (0.08, 0.28) | 0.25 (0.11, 0.39) | - | - |
| Heterogeneity (%) ^3^ | - | 90.3 | 77.5 | 90.0 | - | - |
| Eager (P-value) ^4^ | - | 0.87 | 0.21 | 0.55 | - | - |
| **Abbreviations:** **Sib**, Siblings; **MZ,** Monozygotic twins; **DZ,** Dizygotic twins; **SFA,** Saturated fatty acid; **PUFA**, Poly unsaturated fatty acid.  ^1^ Presented as the number of papers/number of studies.  ^2^ Fisher’s transformed.  ^3^ Not Significant heterogeneities are bolded.  ^4^ Significant publication biases are bolded  ^5^ Percent of energy and grams of intake and servings/day.  ^6^ Percent of energy intake.  ^7^ Grams of intake. | | | | | | |
