# Supplementary material for: Familial resemblance in dietary intake among singletons, twins, and spouses: a meta-analysis of family-based observations
Source: BMC Public Health. 2024 Nov 29;24:3328. doi: 10.1186/s12889-024-20798-x (PMC11605858; doi:10.1186/s12889-024-20798-x)
Supplement: Supplementary file 21 — Supplementary Material 21 [file 12889_2024_20798_MOESM21_ESM.docx]

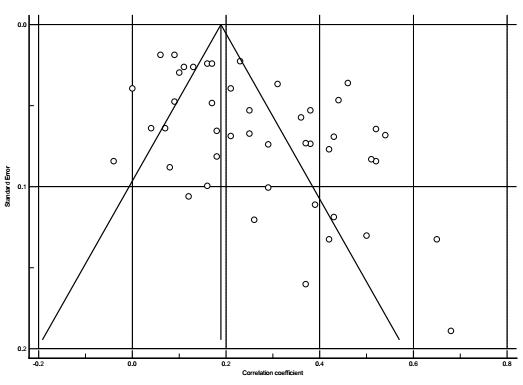


**Supplementary Figure 18.** Funnel plot investigating the potential for publication bias related to total protein intake resemblance among all siblings.
